# Somatostatin receptors 3 and 5 potentiate cholinergic-nerve-mediated contraction in human bronchus

**DOI:** 10.3389/fphar.2025.1677183

**Published:** 2025-11-06

**Authors:** Marion Brollo, Stanislas Grassin-Delyle, Camille Roquencourt, Elisabeth Longchampt, Isabelle Miguet-Besson, Matthieu Glorion, Hélène Salvator, Philippe Devillier

**Affiliations:** 1 Laboratory of Research in Respiratory Pharmacology, VIM Suresnes, UMR-0892, Foch Hospital, Suresnes, France; 2 Faculty of Health Sciences Simone Veil, University of Versailles Saint Quentin en Yvelines, Paris Saclay University, Montigny-leBretonneux, France; 3 INSERM UMR1173 & Mass Spectrometry Platform, University of Versailles Saint Quentin en Yvelines, University Paris Saclay, Montigny-leBretonneux, France; 4 Exhalomics®, Exhaled Breath Analysis Platform, Department of Pulmonology, Foch Hospital, Suresnes, France; 5 Pathology Department, Foch Hospital, Suresnes, France; 6 Department of Thoracic Surgery, Foch Hospital, Suresnes, France; 7 Department of Pulmonology, Foch Hospital, Suresnes, France

**Keywords:** somatostatin receptors, human, lung, bronchus, contraction, immunohistochemistry, cholinergic nerve

## Abstract

**Aim:**

The role of somatostatin (SST) in the modulation of cholinergic neurotransmission has not been explored previously in human bronchi. We investigated the effects of SST, selective agonists of the five SST receptors SSTR, and octreotide (a SSTR_2,3,5_ agonist) on the cholinergic contraction induced *in vitro* either by acetylcholine or by electrical field stimulation (EFS) in human bronchial rings.

**Methods:**

Human bronchial rings (n = 326) were obtained from 32 patients undergoing surgery for lung carcinoma. 5 Hz EFS (biphasic pulse width: 1 ms; constant current: 320 mA for 10 s) induced contractions that reached about ∼30% of the maximum contraction caused by 40 Hz EFS. Bronchial rings were stimulated for 240 min in the presence or absence of various concentrations of SST, octreotide, and selective agonists of each of the five SSTR receptors. Furthermore, the tissue and cellular locations of each of the five types of SSTR was determined by immunohistochemistry.

**Results:**

SST, octreotide, and the SSTR agonists did not change the resting tone or the contractions produced by the cumulative addition of acetylcholine (10^−9^ to 10^−3^ M). In contrast, octreotide and the SSTR_3_ and SSTR_5_ agonists significantly increased the EFS-induced contractions. Immunoreactivity for all SSTR subtypes was detected in the airway’s neural ganglia.

**Conclusion:**

The present study provided new data on the location of SSTR in the human lung: notably, all types of receptor were found in the parasympathetic nerve ganglia of the bronchial wall. We suggest that the activation of prejunctional SSTR_3_ and SSTR_5_ receptors potentiates cholinergic-nerve-mediated contraction induced by EFS in human bronchi.

## 1 Background

The multiple physiological functions and actions of somatostatin (SST) are mediated through five receptors (SSTR_1_-SSTR_5_). These receptors belong to the G–protein–coupled receptor family and are involved in several distinct signal transduction pathways (Günther et al., 2018). The gene coding for SSTR_2_ (*SSTR2*) is subject to alternate mRNA splicing and thus produces two receptor isoforms (SSTR_2A_ and SSTR_2B_), which differ only with regard to the number of amino acids and the amino acid composition of the cytoplasmic carboxy-terminal tail (Günther et al., 2018).

The SSTRs’ expression and (in particular) functions in the human lung have not been extensively characterized. With regard to protein expression, all the SSTRs other than SSTR_2A_ have been found on cells in the bronchial glands (Taniyama et al., 2005). Furthermore, SSTR_2A_ and SSTR_4_ have been found on bronchial, bronchiolar and alveolar epithelial cells (Gugger et al., 2004; Borie et al., 2008; Varecza et al., 2009), SSTR_2B_, SSTR_4_ and SSTR_5_ are present on alveolar macrophages, and SSTR_4_ has been identified on smooth muscle and vascular endothelial cells (Borie et al., 2008; Varecza et al., 2009; Günther et al., 2018). SST is known to inhibit lipopolysaccharide-induced cytokine production by human lung macrophages, although the receptors involved have not been identified (Balibrea et al., 1994). Furthermore, SSTR_2A_ receptors might be involved in the inhibition of pulmonary fibrosis (Borie et al., 2008). All the other data on SST’s effects in the lung come from animal studies. In rodents, SST and its synthetic analogues inhibited endotoxin- and ovalbumin-induced airway inflammation and the resulting bronchial hyperreactivity (Helyes et al., 2006; Helyes et al., 2007; Helyes et al., 2009; Elekes et al., 2008). These effects involved (at least in part) the activation of SSTR_4_ on bronchial smooth muscles and the prejunctional SSTR_4_ that mediates the inhibition of neuropeptide release from afferent sensory nerves (Helyes et al., 2006; Elekes et al., 2008). Although the vast majority of SST’s effects are inhibitory (particularly for neurogenically mediated contractile responses (Pintér et al., 2006; Günther et al., 2018), direct contractile effects have been described with several isolated preparations: guinea pig isolated ileum and vas deferens (Feniuk et al., 1993), human saphenous veins (Dimech et al., 1995) and colonic smooth muscle cells (Corleto et al., 2006). Furthermore, SST has been shown to potentiate cholinergic neurotransmission in isolated ferret trachea (Sekizawa et al., 1989). On the other hand, adverse effects such as dyspnea or bronchospasm have been reported in clinical trials of somatostatin and octreotide, a stable SSTR2, SSTR3 and SSTR5 agonist (see the summary of product characteristics).

The objectives of the present study were to (i) describe the expression of the various SSTRs in the human lung more fully, and (ii) analyze the direct and indirect effects of SST, octreotide, and other SSTR-specific agonists on isolated human bronchial preparations.

## 2 Methods

Our hospital’s pathology department supplied lung tissue from macroscopically healthy parts of the lungs of 32 patients (26 males and 6 females; mean ± standard deviation (SD) age: 63.8 ± 8.7 years; 11 current smokers, 18 former smokers, and 3 never-smokers; mean ± SD pack-years: 37.5 ± 19.8; mean ± SD FEV1 = 78.8 ± 15.1%; mean ± SD FEV1/FVC ratio: 0.81 ± 0.15. Four of the patients had non-severe COPD. All the patients had undergone surgical resection for lung carcinoma and had not received chemotherapy or radiotherapy before surgery. The use of human lung tissue for *in vitro* experiments was approved by the local independent ethics committee (Comité de Protection des Personnes Ile de France VIII, Boulogne-Billancourt, France; reference: CPP8 - DC 11 10 05). Each patient gave their informed consent to the use of surgical samples for research purposes.

### 2.1 Immunohistochemical detection of the SST receptors

Lung tissue samples were obtained from five patients who had all stopped smoking for at least 3 years. The samples were fixed in 4% buffered formaldehyde within 1 hour after lobectomy, embedded in paraffin, and cut into 4 µm serial sections. The tissue sections were then dewaxed in xylene and rehydrated with ethanol. Before the immunohistochemical procedure, the antigens were retrieved by incubating the tissue sections in a boiling water canner for 20 min. Non-specific binding of the secondary antibody was prevented by incubation with normal goat serum for 1 h. The slides were stained with an automated immunohistochemistry system (Ventana NexeS IHC, Roche Diagnostics, Meylan, France), incubated with human SSTR subtype-specific rabbit polyclonal antibodies (Gramsch Laboratories/Biotrend, Köln, Germany) (Taniyama et al., 2005) at an optimal dilution/concentration ratio and optimal incubation time (SS-840 (anti-SSTR_1_): 1/200, 22 min; SS-800 (anti-SSTR_2A_) 1/200, 22 min; SS-860 (anti-SSTR_2B_): 1/200 22 min; SS-850 (anti-SSTR_3_): 1/200, 32 min; SS-880 (anti-SSTR_4_): 2 μg/mL, 3 h; and SS-890 (anti-SSTR_5_): 1/200, 32 min)) and then processed with a rabbit-specific horseradish peroxidase/3,3′-diaminobenzidine kit (RUO, Roche Diagnostics, Meylan, France). The specificity of the immunohistochemical reaction was confirmed by the absence of a positive signal after the secondary antibody was used without the primary antibody. The positive control experiments involved normal human pancreatic parenchyma and/or pulmonary carcinoid tumour tissue.

### 2.2 Experiments on rings of isolated human bronchus

The bronchi (mean ± SD inner diameter: 1.9 ± 0.5 mm) were dissected free from adhering lung parenchyma and connective tissue and then cut into rings of 4–7 mm in length. Eight to 24 rings were obtained from a given patient’s sample and then used as paired preparations. Before use, the rings were stored overnight at +4 °C in a Krebs-Henseleit solution (KHS, in mM: NaCl 119, KCl 5.4, CaCl_2_ 2.5, KH_2_PO_4_ 1.2, MgSO_4_ 1.2, NaHCO_3_ 25, glucose 11.7) equilibrated with O_2_/CO_2_ (95:5). On the following day, human bronchial segments were placed in an isolated organ bath filled with 5 mL of KHS, oxygenated with O_2_/CO_2_ (95:5) and thermostated at +37 °C (pH 7.4). No peptidase inhibitors were used. Tension was measured isometrically with a strain gauge (UF1; Piodem, Canterbury, Kent, United Kingdom) connected to an amplifier (EMKA Technologies, Paris, France). Data were acquired, processed and analyzed with a computerized system running IOX v1.56.8 and Datanalyst v1.58 softwares (EMKA Technologies). In all experiments, the bronchial rings were suspended with an initial load of 2 g and equilibrated for 60–90 min. The KHS in the bath was changed every 15–20 min. At the end of the equilibration period, the resting load was stable at ∼1–3 g. As described previously, bronchi were first contracted maximally with acetylcholine (ACh, 3 mM) and then washed and equilibrated again for 60 min before the experimental procedures were initiated (Grassin-Delyle et al., 2014; Heusler et al., 2015; Naline et al., 2018). A total of 326 rings were used in the experiments on isolated rings of human bronchus. The ranges of agonist concentrations employed here were chosen to maximize the discrimination between the various SSTRs, based on the literature data (Rohrer et al., 1998; Patel, 1999; Rohrer and Schaeffer, 2000; Günther et al., 2018).

#### 2.2.1 Effects on basal tone and the contractile responses to exogenous ACh

Each agonist’s effect on a ring’s basal tone was assessed by cumulative addition (0.1 nM–1 μM, in logarithmic increments) at 15 min intervals. To investigate the agonist’s respective effects on ACh- or KCl-induced contractions, a first cumulative concentration-response curve (CRC) was obtained for ACh (10 nM–1 mM) or KCl (1 mM–1 M). After extensive washing and equilibration for 1 h, the rings were incubated with the agonist for 10 min prior to the measurement of a second cumulative CRC for ACh or KCl (Advenier et al., 1986; Naline et al., 2018). The CRC-derived data on efficacy and potency were expressed as Emax and–log EC_50_ (pD_2_), respectively.

#### 2.2.2 Electrical field stimulation–induced contraction of human bronchus

In order to trigger the neural release of ACh, electrical field stimulation (EFS) experiments were performed as described previously (Grassin-Delyle et al., 2014; Naline et al., 2018). EFS was performed in organ baths fitted with two platinum plate electrodes placed alongside the tissue (10 mm apart) and connected to a stimulator (EMKA Technologies). A stimulation train consists in biphasic square-wave pulses with a constant current of 320 mA and a pulse duration of 1 ms delivered for 10 s at 5 Hz. The EFS-induced contractions at frequencies ranging from 5 to 40 Hz in paired human bronchi samples with inner diameters ranging from 1 to 3 mm were similar (Naline et al., 2018). These contractions were fully blocked by atropine (1 µM) and tetrodotoxin (1 µM); this demonstrated the involvement of ACh release from cholinergic nerves, as shown previously in our laboratory (Grassin-Delyle et al., 2014; Naline et al., 2018) and in others (Watson et al., 1998). For the EFS experiments, the cyclo-oxygenase inhibitor indomethacin (1 µM), and the cysteinyl leukotriene antagonist MK476 (1 µM) were added to the KHS at the beginning of the experiments; this respectively avoided the influence of leukotrienes and prostaglandins on the neuronal responses (Watson et al., 1998; Naline et al., 2007; Naline et al., 2018; Grassin-Delyle et al., 2014). At the end of the equilibration period, the resting load was stable at ∼1 g. Eight to 16 bronchial rings were simultaneously tested, along with at least one time-control preparation per series of 8 rings (to assess the response for the duration of the experiments). To assess each preparation’s baseline response, a first train of EFS was applied twice at 10 min intervals. This stimulation (at 5 Hz, as used by Sekizawa et al. (1989)) caused a contraction corresponding to 32% ± 6% of the maximum response to 40 Hz stimulation (n = 8), in order to leave room for an increase in EFS-induced contraction (Sekizawa et al., 1989; Fernandes et al., 1999; Grassin-Delyle et al., 2014; Naline et al., 2018). SST, the SSTR agonists or vehicle was added to the bath 10 min before the beginning of a second train of stimulations (delivered every 10 min for 1 h and then every 20 min for 3 h). A given ring was used to study only one concentration of one compound (Grassin-Delyle et al., 2014; Naline et al., 2018).

### 2.3 Drugs

SST (UCB, Nanterre, France) and octreotide (Sandostatin™, Novartis Pharma, Rueil-Malmaison, France) were obtained as solutions for injection. The SSTR_1_ agonist CH-275 (Des-AA1,2,5-[DTrp8,IAmp9]-SRIF) was purchased from Neo MPS (Strasbourg, France) (Rivier et al., 2001). The other selective SSTR agonists (SSTR_2_: L-779,976; SSTR_3_: L-796,778; SSTR_4_: L-803,087; SSTR_5_: L-817,818) were kindly provided by Dr Susan P. Rohrer (Merck Research Laboratories, Rahway, NJ, United States). L-796,778 is a partial agonist for SSTR_3_ (Rohrer et al., 1998; Rohrer and Schaeffer, 2000). The SSTR agonists’ selectivity is described in Supplementary Table S1. All the agonists were dissolved to a concentration of 10^−2^ M in DMSO, and subsequent dilutions were prepared in assay buffer. ACh and atropine were obtained from Sigma (St Louis, MI, United States).

### 2.4 Data analysis

The experimental data are quoted as the mean ± standard error of the mean (SEM); the n represents the number of donors. For each bronchial ring, the values were expressed as the percentage of the contraction obtained with either the maximum concentration of ACh or during the initial EFS train.

A one- or two-way repeated-measures analysis of variance (ANOVA) and then Dunnett’s post-test for multiple comparisons were used to assess the EFS data. The effects of agonist concentration and stimulation time were evaluated in a two-way ANOVA. The EFS data were also evaluated in a one-way ANOVA after calculation of the areas under the concentration-time curves (AUC), using the linear trapezoidal method. The agonists’ effects on the CRC (E_max_ and pD_2_) for ACh or KCl were analyzed in a one-way ANOVA. The threshold for statistical significance was set to p < 0.05. All analyses were performed using GraphPad Prism software (version 8.4.2, GraphPad Software Inc., San Diego, CA, United States).

## 3 Results

### 3.1 Immunohistochemical detection of the SST receptors on human bronchus and lung parenchyma

**FIGURE 1:**
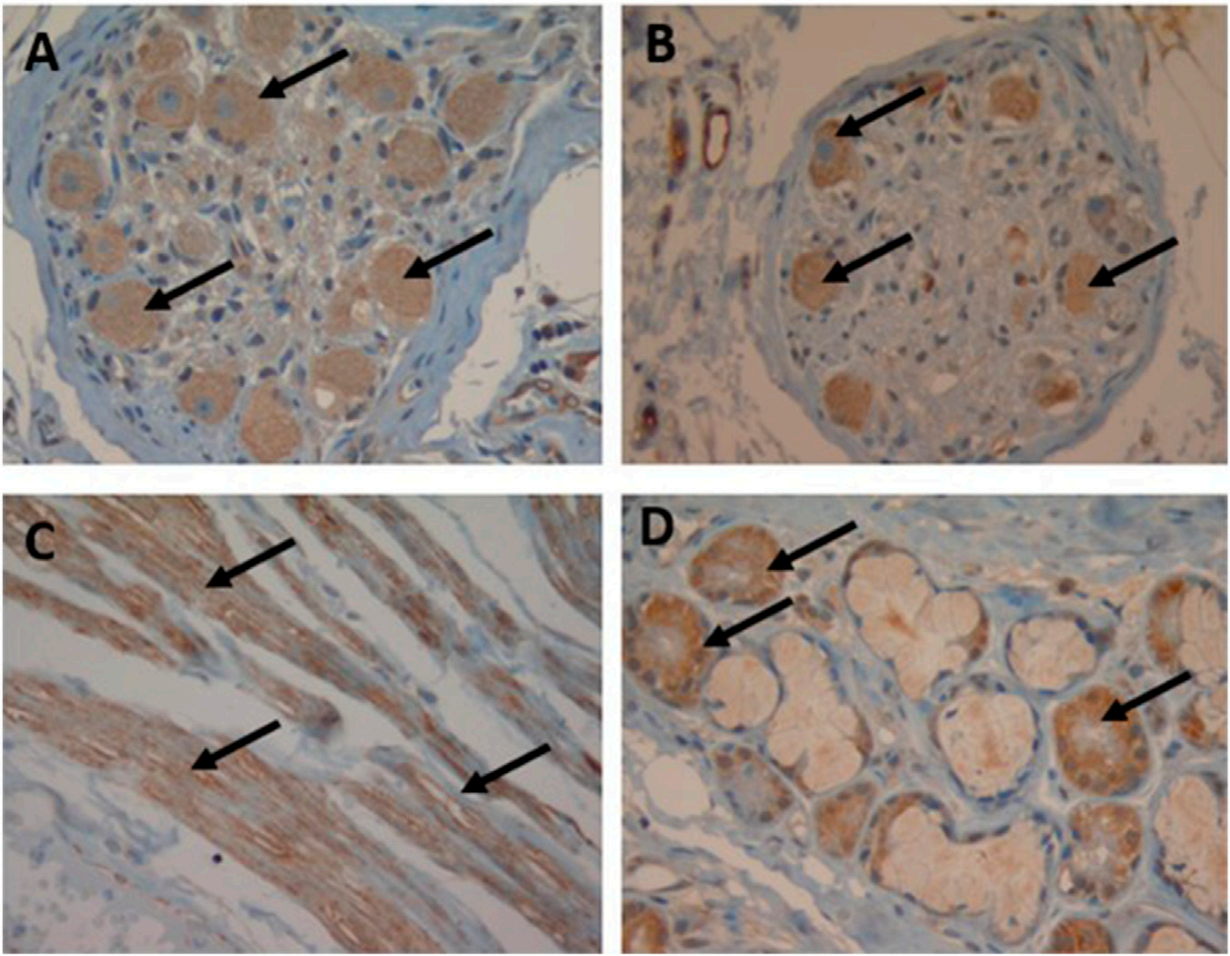
Immunohistochemical localization of SST receptors in the human bronchus. Arrows indicate SST receptor positivity (magnification: 40x): neural ganglia staining with SSTR_3_ **(A)** and SSTR_5_ **(B)**, smooth muscle staining with SSTR_4_ **(C)** and submucosal gland staining with SSTR_1_ **(D)**.

In the bronchus, all the SSTRs were expressed on the parasympathetic ganglia (rank order: SST_4_ > other SSTRs) and the submucosal glands (rank order: SSTR_1,2A,2B,3,5_ > SSTR_4_). On smooth muscle, SSTR_4_ and (to a lesser extent) SSTR_2A_, SSTR_3_ and SSTR_5_ were expressed but SSTR_1_ and SSTR_2B_ were not (Table 1; Figure 1). In the parenchyma, all the SSTRs other than SSTR_4_ were expressed by bronchiolar epithelial cells and some pneumocytes. Lastly, all the SSTRs other than SST_2A_ and SST_4_ were expressed by alveolar macrophages (Table 1).

**TABLE 1:** The relative abundance of SST receptor immunoreactivity in human bronchus and lung parenchyma.

	SSTR_1_	SSTR_2A_	SSTR_2B_	SSTR_3_	SSTR_4_	SSTR_5_
Neural parasympathetic ganglia	+	+	+	+	++	+
Submucosal glands	++	++	++	++	+	++
Smooth muscle	-	+	-	+	++	+
Bronchiolar epithelial cells	+	+	+	+	-	+
Alveolar macrophages	+	-	+	+	-	+
Pneumocytes	(+)	(+)	+	(+)	-	+

The intensity of immunolabeling was scored ++ when it was of the same intensity as in the positive controls (normal human pancreatic parenchyma and/or pulmonary carcinoid tumour tissue). Obvious but less intense immunostaining was scored as +, while immunostaining that was found inconsistently or weakly was scored as (+).

### 3.2 Effect of SST and SSTR agonists on the EFS-induced contraction of human bronchus

**FIGURE 2:**
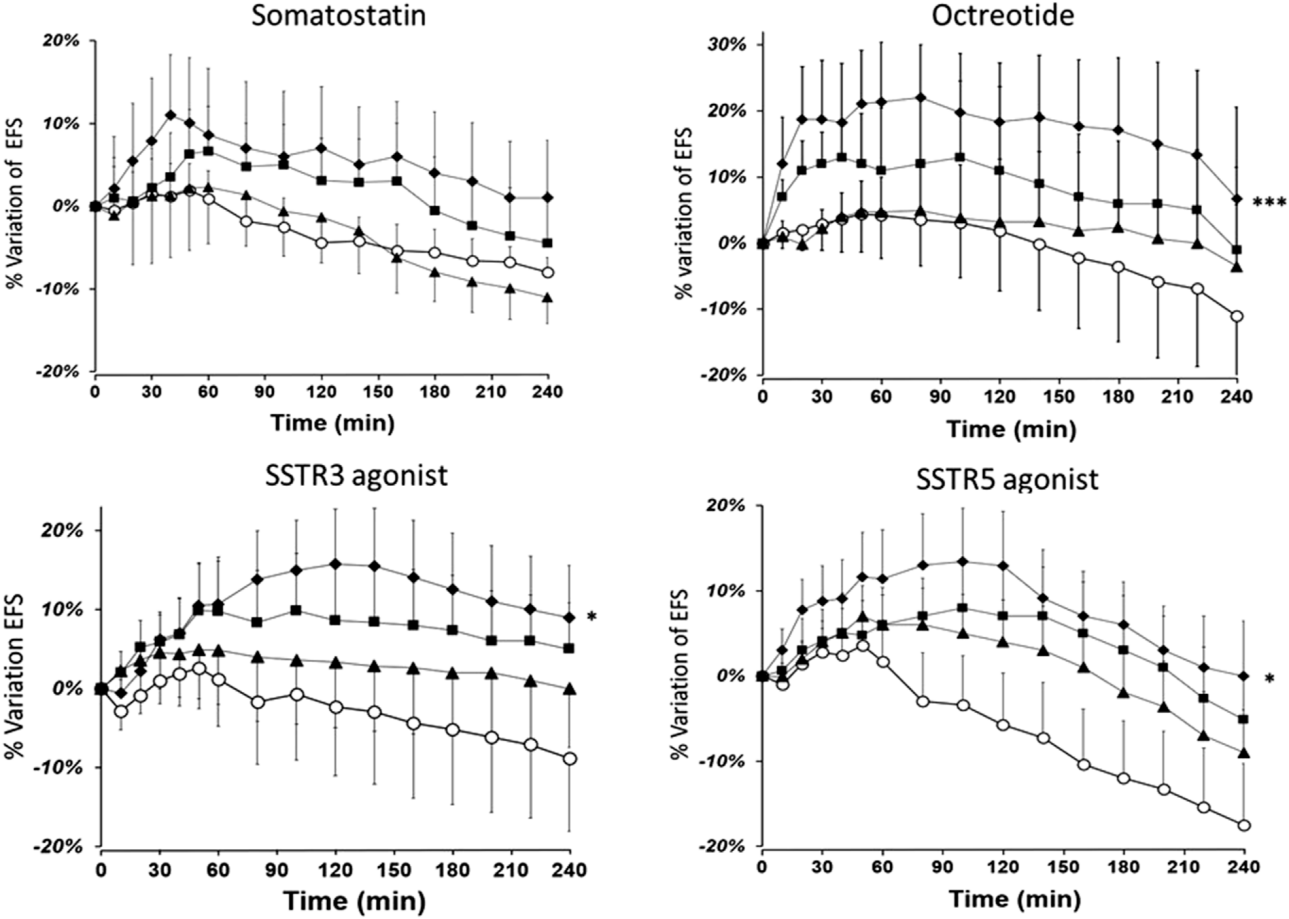
The time course of the enhancing effect of octreotide and the SSTR3 and SSTR5 agonists on the EFS-induced contraction of human bronchial rings. The data are quoted as the mean ± SEM percentage of EFS-induced contraction of paired bronchial rings from 9 to 10 patients. (O: controls; ▲10^−9^ M, ■: 10^−8^ M, and ◆10^−7^ M for octreotide and the SSTR5 agonist; ▲10^−8^ M, ■: 10^−7^ M, and ◆ 3 × 10^−7^ M for the SSTR3 agonist), *: P < 0.05, ***: P < 0.001).

SST at a concentration of 10^−7^ M induced a small (∼10%, p = 0.06) increase in EFS-induced contraction. The increase in EFS-induced contraction caused by octreotide was concentration-dependent. At the optimal concentration of 10^−7^ M, octreotide caused a significant increase in EFS-induced contraction versus the control; the mean maximum increase was 22% ± 8% (p < 0.05) at the 50th minute (Figure 2). The mean increase in the AUC was also significant with octreotide 10^−7^ M (33% ± 5%*min, P < 0.001).

Furthermore, the optimal concentrations of the SSTR_3_ (3 × 10^-7^ M) and SSTR_5_ (10^−7^ M) agonists caused a significant increase in EFS-induced contraction. The mean maximum increases induced by the SSTR_3_ and SSTR_5_ agonists were 16% ± 4% and 19% ± 5% after 90 and 120 min of stimulation, respectively. At the optimal concentrations, the mean increases in the AUC were also significant (19% ± 6%*min, p = 0.012 for SSTR_3_, and 22% ± 7%*min, p = 0.013 for SSTR_5_). In contrast, the agonists of the other SSTR receptors (SSTR_1_, SSTR_2_, and SSTR_4_) did not alter EFS-induced contraction (n = 6–8) (Supplementary Figure S1).

### 3.3 Effect of SST and SSTR agonists on the resting tone and the acetylcholine-induced contraction of human bronchus

Neither SST, octreotide, nor any of the SSTR agonists was associated with a change in the resting tone. The contractile responses to the cumulative addition of increasing concentrations of ACh were not modified by pre-incubation of the rings with SST, octreotide, or the SSTR agonists, since there was no shift in the CRC and no significant difference in the maximum response (Table 2; Supplementary Figure S2). Since SSTRs can inhibit voltage-operated calcium channels, we also tested the effects of octreotide and SSTR_3_ and SSTR_4_ agonists on KCl-induced contraction (Advenier et al., 1984; Advenier et al., 1986). None of these three SSTR agonists altered KCl-induced contraction (n = 5-6, data not shown).

**TABLE 2:** The effects of the SSTR agonists on the contraction induced by exogenous ACh (n = 5–6). The concentrations of SSTR agonists used here were either the optimal concentration with respect to the effect on the EFS-induced contraction (for octreotide (0.1 µM), the SSTR_3_ agonist (0.3 µM) and the SSTR_5_ agonist (0.1 µM)) or the maximum concentration tested (for somatostatin and the other SSTR agonists). The data are quoted as the mean ± SEM.

	*E*_max_ (%)	pD2
Vehicle	100	5.12 ± 0.16
Somatostatin	97.8 ± 2.1	5.13 ± 0.12
Octreotide	95.6 ± 3.4	5.17 ± 0.15
SSTR_1_ agonist	98.4 ± 1.8	5.19 ± 0.20
SSTR_2_ agonist	98.1 ± 1.7	5.09 ± 0.15
SSTR_3_ agonist	98.3 ± 1.4	5.03 ± 0.20
SSTR_4_ agonist	99.3 ± 1.1	5.21 ± 0.16
SSTR_5_ agonist	99.7 ± 1.3	5.15 ± 0.19

## 4 Discussion

Our study generated new data on the locations of SST receptors in the human lung. We notably found that in the bronchial wall, all types of receptor were expressed in the parasympathetic nerve ganglia. In bronchial smooth muscle, only SSTR_1_ and SSTR_2B_ receptors were not expressed. We suggest that in human bronchi, (i) the activation of prejunctional SSTR_3_ and SSTR_5_ receptors potentiates the cholinergic-nerve-mediated contraction induced by EFS, and (ii) neither SSTR has a direct effect on muscle tone or acetylcholine-induced contraction.

Our present results confirmed the report by Taniyama et al. (2005) and showed that all types of SSTR are indeed expressed by bronchial submucosal glands. It has been reported that human tissues contain only the SSTR_2A_ variant, whereas both SSTR_2A_ and SSTR_2B_ have been identified in rodent tissues (Günther et al., 2018). However, expression of SSTR_2A_ and SSTR_2B_ has been reported in human tissues, including thyrotropin-secreting pituitary adenomas, thyroid tumours (Pisarek et al., 2015; Thodou and Kontogeorgos, 2020), and the lung (alveolar macrophages (SSTR_2B_), pneumocytes, submucosal glands) (Taniyama et al., 2005; Borie et al., 2008). In the present study, the two SSTR_2_ forms were found on the neural ganglia, the submucosal glands, the bronchiolar epithelial cells and the pneumocytes, while SSTR_2A_ was expressed by smooth muscle and SSTR_2B_ was expressed by alveolar macrophages. SSTR_2B_ receptor expression is low in normal lung parenchyma but is reported elevated in the fibrotic lung (particularly on epithelial cells) (Borie et al., 2008). Our results suggest that alveolar macrophages express SSTR_1_ and SSTR_3_ in addition to SSTR_2B_ (Borie et al., 2008) and SSTR_5_ (Günther et al., 2018).

We did not detect SSTR_4_ on bronchiolar epithelial cells (which nevertheless expressed all the other SSTRs) or macrophages (in contrast to the report by Varecza et al. (2009)). However, Varecza et al. mainly observed SSTR_4_ labelling of macrophages in samples from inflamed lungs (pneumonia and bronchiectasis). To the best of our knowledge, only SSTR_4_ expression has been documented in human bronchial smooth muscle (Varecza et al., 2009). We also found that SSTR_2A_, SSTR_3_ and SSTR_5_ were expressed by human bronchial smooth muscle.

SST is present in enteric neurons and SSTR_1_, SSTR_2_, and SSTR_3_ receptors are expressed on enteric ganglia and the corresponding nerve fibres and nerve terminals (Günther et al., 2018). Given that the airways’ nerves are derived embryologically from those of the gut, the presence of SSTR in the airway ganglia was not unexpected (Barnes, 1990). Indeed, we found that all the SSTR were expressed by the airway neural ganglia. In the present study, lung tissue was obtained from former smokers who had undergone surgery for cancer, which limits the extrapolation of our findings on SSTR receptor localization to healthy human lungs.

To the best of our knowledge, the present study is the first to have studied the effects of SST and SSTR agonists on the human bronchus. Neither SST, octreotide, nor any of the other SSTR agonists changed the resting tone or maximum response or shifted the CRC for ACh or KCl. The absence of direct effects is in line with a report on the ferret trachea, in which SST did not significantly change the resting muscle tone or the CRC for ACh (Sekizawa et al., 1989).

With regard to human smooth muscles other than those of bronchial origin, SSTR_1_, SSTR_2_, and SSTR_4_ are expressed in the aorta, the internal mammary artery, and the saphenous vein (Curtis et al., 2000), whereas SSTR_2_ is involved in the contraction of isolated saphenous vein (Dimech et al., 1995). In the gastrointestinal tract, the circular smooth muscle expresses SSTR_2_ only and the longitudinal smooth muscle expresses SSTR_1_, SSTR_2_, and SSTR_3_ (Corleto et al., 2006). SST exerted a relatively weak contractile effect (around a third of that of carbachol) on the circular and longitudinal human colonic smooth muscle cells; this effect was thought to be mediated by the activation of SSTR_1_ and SSTR_2_ (Corleto et al., 2006). However, SST’s main effect was SSTR_2_-mediated relaxation of carbachol-induced contraction (Corleto et al., 2006).

Most of the best-characterized actions of the SST receptors are inhibitory. Activation of Gi/Go proteins by SST caused a reduction in cAMP by inhibition of adenylyl cyclase, and inhibition of calcium influx through voltage-operated calcium channels (Günther et al., 2018). These inhibitory effects are additive in excitable cells, such as neurons (Günther et al., 2018). However, it has been shown that SST stimulated the release of ACh in the myenteric and submucous plexus of the guinea pig ileum (Yau et al., 1983; Lu et al., 1990). More specifically, SST potentiated EFS-induced contraction of the ferret trachea, although the researchers did not determine which SSTR were involved (Sekizawa et al., 1989). Our results suggest that SSTR agonists potentiate EFS-induced contraction of the human bronchus. SST’s weak modulatory effect on EFS-induced contraction in the present study is probably related to the molecule’s short half-life–a drawback that limits its therapeutic use (Günther et al., 2018). Accordingly, two stable SST analogues (octreotide and lanreotide) have been developed for clinical use (Chen et al., 2023). Octreotide is highly selective for SSTR_2_, SSTR_3_ and SSTR_5_ (Supplementary Table S1). We found that SSTR_3_ and SSTR_5_ agonists potentiated EFS-induced contraction, whereas an SSTR_2_ agonist had no effect on EFS-induced contraction. The apparently greater effect of octreotide on EFS-induced contraction is probably explained by the dual stimulation exerted by this agonist on the SSTR3 and SSTR5 receptors and by the partial agonist activity of the SSTR3 agonist. Given that SSTR are definitely present on bronchial parasympathetic nerve ganglia and probably present on post-ganglionic nerve fibres, our results suggest that stimulation of the SSTR_3_ and SSTR_5_ receptors potentiates the cholinergic-nerve-mediated contraction induced by EFS in human bronchi via presynaptic mechanisms. Our findings may help explain the occurrence of dyspnea or bronchospasm during treatment with somatostatin and octreotide.

Very few mediators have been shown to potentiate cholinergic neurotransmission in the human bronchus. Along with SST, endothelin-1 was found to potentiate EFS-mediated contraction in the human bronchus via activation of cholinergic nerve ET_A_ and ET_B_ receptors (Fernandes et al., 1999). The amplitude of the endothelin-potentiating effect ( ~ 20%) was of the same order as that of octreotide and the SSTR_3_ and SSTR_5_ agonists (Fernandes et al., 1999; D’Agostino et al., 2001). Even though neurokinins can facilitate cholinergic neurotransmission in the rabbit airways (via NK_1_ and NK_2_ receptors) and guinea pig airways (via the NK_1_ receptor), they have no demonstrable effects on human airways (Belvisi et al., 1994).

## 5 Conclusion

The present study provided new data on the location of SST receptors in the human lung. Our results suggest that the activation of prejunctional SST_3_ and SST_5_ receptors potentiates the cholinergic-nerve-mediated contraction induced by EFS in human bronchi.

## Data Availability

The raw data supporting the conclusions of this article will be made available by the authors, without undue reservation.
