# Supplementary material for: Somatostatin receptors 3 and 5 potentiate cholinergic-nerve-mediated contraction in human bronchus
Source: Front Pharmacol. 2025 Nov 6;16:1677183. doi: 10.3389/fphar.2025.1677183 (PMC12631201; doi:10.3389/fphar.2025.1677183)

Table S1: selectivity of the agonists for the various somatostatin receptor subtypes

| **Compound** | **SSTR_1_** | **SSTR_2_** | **SSTR_3_** | **SSTR_4_** | **SSTR_5_** |
| --- | --- | --- | --- | --- | --- |
| Somatostatin-14 (K_i_) | 0.4 | 0.04 | 0.7 | 1.7 | 2.3 |
| Octreotide (K_i_) | >1,000 | **0.4** | **4.4** | > 1,000 | **5.6** |
| CH-275 (IC_50_) | **33** | > 10,000 | 345 | > 1,000 | > 10,000 |
| L-779,976 (K_i_) | 2,760 | **0.05** | 729 | 310 | 4,260 |
| L-796,778 (K_i_) | 1,255 | > 10,000 | **24** | 8,650 | 1,200 |
| L-803,087 (K_i_) | 199 | 4,720 | 1,280 | **0.7** | 3,880 |
| L-817,818 (K_i_) | 3.3 | 52 | 64 | 82 | **0.4** |

The selectivity is expressed as the IC_50_ or K_i_ (nM). The data on octreotide are from Patel et al. (1999) and Gunther et al. (2018). The data on the SSTR_1_ agonist CH-275 (Des-AA1,2,5-[DTrp8,IAmp9]-SRIF) are from Rivier et al. (2001). The data on the selective agonists (SSTR_2_: L-779,976; SSTR_3_: L-796,778; SSTR_4_: L-803,087; SSTR_5_: L-817,818) are from Rohrer et al. (1998 & 2000). L-796,778 is a partial agonist of SSTR_3_.

Figure S1: Time course of the effect of somatostatin and the SSTR_1_, SSTR_2_ and SSTR_4_ agonists on the EFS-induced contraction of human bronchial rings. The data are quoted as the mean ± SEM percentage of EFS-induced contraction of paired bronchial rings from 7-10 patients. (O: controls; ▲10^-9^ M, ◼: 10^-8^ M, and ◆10^-7^ M).


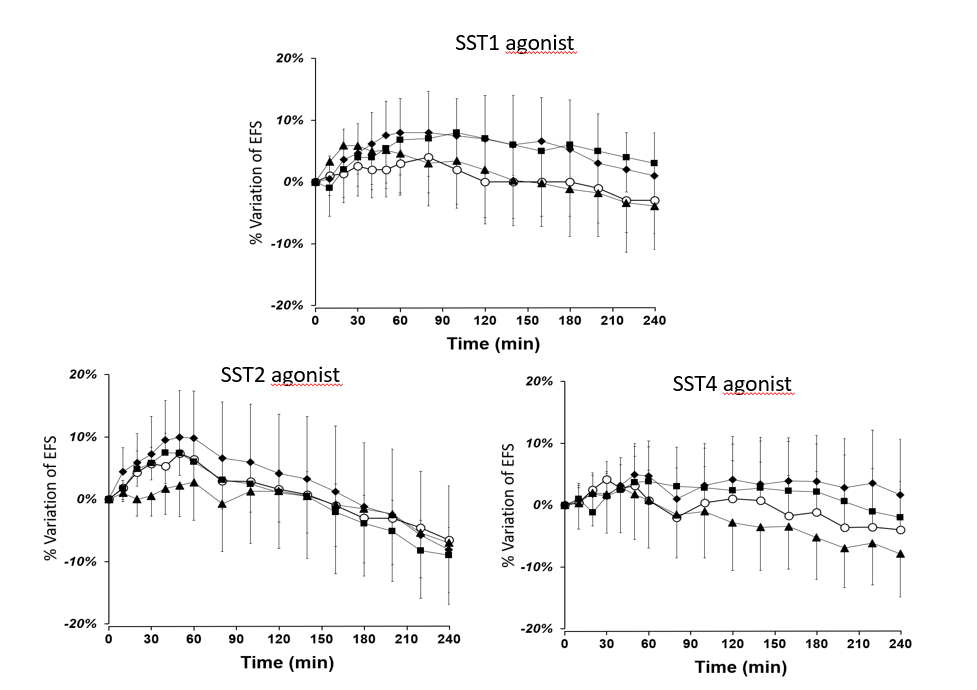


Figure S2: Effects of octreotide (0.1 µMM, ◼), the SSTR_3_ agonist (0.1 µMM, ◆) and the SSTR_5_ agonist (0.3 µM, ▲) on the ACh-induced contraction (⭘) of paired human bronchial rings (n=6). The data are quoted as the mean ± SEM percentage of the maximum control contraction with ACh.


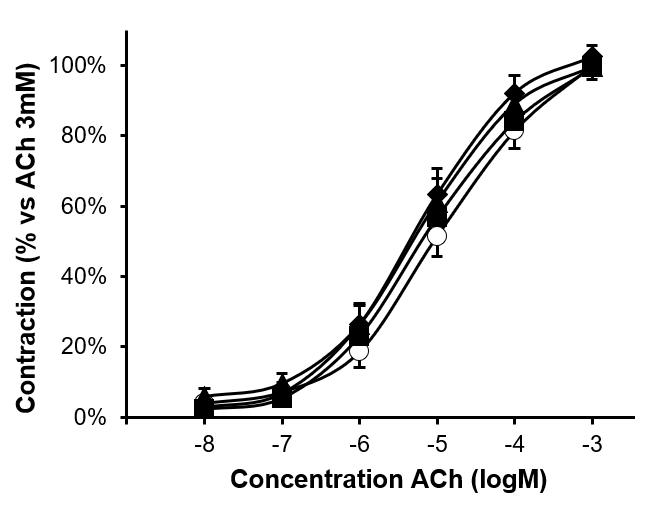

Supplement: Supplementary file 1 [file Supplementaryfile1.docx]
